# Antibacterial and COX-1 Inhibitory Effect of Medicinal Plants from the Pamir Mountains, Afghanistan

**DOI:** 10.3390/plants1020074

**Published:** 2012-10-24

**Authors:** Anne S. Jeppesen, Jens Soelberg, Anna K. Jäger

**Affiliations:** 1 Department of Drug Design and Pharmacology, Faculty of Health and Medical Sciences, University of Copenhagen, 2 Universitetsparken, 2100 Copenhagen, Denmark; Email: annesjeppesen@gmail.com; 2 NaturMedicinsk Museum, Faculty of Health and Medical Sciences, University of Copenhagen, 2 Universitetsparken, 2100 Copenhagen, Denmark; Email: jenssoelberg@gmail.com

**Keywords:** Afghanistan, antibacterial, COX, medicinal plants, Pamir

## Abstract

Plants used to treat inflammatory ailments, pain, fever and infections in the Pamir Mountains in northeastern Afghanistan, were tested for antibacterial and COX-1 inhibitory activity. Water and ethanol extracts of 20 species were tested for antibacterial activity against two gram positive and two gram negative bacteria. The ethanol extract of *Arnebia guttata* inhibited *Staphylococcus aureus* with a MIC of 6 µg/mL. Water and ethanol extracts of *Ephedra intermedia *and the ethanol extracts of *Lagochilus cabulicus* and *Peganum harmala* inhibited *Staphylococcus aureus* at 0.5 mg/mL,and the *P. harmala* extract further inhibited the growth of *Bacillus subtilis * and *E. coli*, also with MICs of 0.5 mg/mL. Ethanol extracts of *Artemisia persica* (IC_50_: 0.5 µg/mL), *Dragocephalum paulsenii *(IC_50_: 0.5 µg/mL), *Ephedra intermedia *(IC_50_: 3.8 µg/mL), *Hyoscyamus pusillus*, *Nepeta parmiriensis* (IC_50_: 0.7 µg/mL) and *Rumex patientia *subsp*. pamiricus* (IC_50_: 3.5 µg/mL) exhibited COX-1 inhibitory activity. The observed *in vitro* activities support the use of some of the plant species in the traditional medicine systems of the Pamir Mountains.

## 1. Introduction

The Wakhan Corridor, in the Pamir Mountains of North-eastern Afghanistan is populated by the Wakhi and Kyrgyz peoples. This area is one of the most remote and isolated areas in the world and the populations rely almost solely on their local herbal medicine. The Third Danish Pamir Expedition documented plant use by both people. A number of plants used to treat infectious diseases, fever and pain was recorded.

The flora of the Pamir/Hindukush Mountains is in general related to the Tibetan and Central Asian floras [1]. Some of the species, however, have a fairly broad distribution through alpine Eurasia, and are well described. Others are endemic to the Pamir/Hindukush and the investigation of these plants has been neglected. All species considered in this study are adapted to a dry high-altitude steppe-environment or associated with man-made irrigation in Wakhi villages, and are all reasonably common within the Wakhan Corridor.

The present study investigated antibacterial and cyclooxygenase-1 (COX-1) inhibitory activity of plants from the Pamir Mountains. 

## 2. Results and Discussion

### 2.1. Testing for Antibacterial Activity

Water and ethanol extracts of 20 species used in the Pamir Mountains for ailments which could be caused by bacterial infections, were investigated for antibacterial activity against two gram positive and two gram negative bacteria. Most of the tested extracts did not inhibit the test bacteria.

Water and ethanol extracts of *Ephedra intermedia * and the ethanol extracts of *Lagochilus cabulicus* and *Peganum harmala* inhibited *Staphylococcus aureus* at 0.5 mg/mL,and the *P. harmala* extract further inhibited the growth of *Bacillus subtilis *and *E. coli*, also with MICs of 0.5 mg/mL (Table 1). Antibacterial activity of *P. harmala* has been demonstrated previously [2], the activity is due to harmane-type alkaloids [3]. The best antibacterial activity was obtained with the ethanol extract of *Arnebia guttata*, with an exceptional low MIC of 6 µg/mL against *S. aureus*, very close to the value of 2 µg/mL obtained with streptomycin (Table 1). The water extract of *A. guttata* did not show activity. When used in the Pamir Mountains, the root material is finely chopped and then fried in oil, and the oil is then applied to cotton wool and inserted in the outer ear against earache. This preparation makes sense as it seems the active compounds are not extracted with water. *Arnebia* species have been used from Turkey to China to treat various bacterial infections [4,5]. The antibacterial activity of *Arnebia* species is due to alkannin and derivatives thereof [5]. *A. guttata* had a strong red color, indicating the presence of alkannin-derivatives, and previously several of such compounds have been shown to be present in the species [6,7].

### 2.2. Testing for COX-1 Inhibition

A number of the recorded uses of the plants indicated that the plants might inhibit the prostaglandin biosynthesis, and thereby act as anti-inflammatories, pain killers or febrifuges. Ten plant species were tested for COX-1 inhibitory activity. Ethanol extracts of *Artemisia persica *(IC_50_: 0.5 µg/mL), *Dragocephalum paulsenii* (IC_50_: 0.5 µg/mL), *Ephedra intermedia* (IC_50_: 3.8 µg/mL), *Hyoscyamus pusillus*, * Nepeta parmiriensis* (IC_50_: 0.7 µg/mL)and *Rumex patientia *subsp*. pamiricus* (IC_50_: 3.5 µg/mL) exhibited the best COX-1 inhibitory effect (Table 2).

**Table 1:** Antibacterial activity of plant species used in the Pamir Mountains to treat ailments related to bacterial infections.

Plant species	Family	Voucher No. (J. Soelberg)	Reported use	Used part	MIC (µg/mL)			
					*S. aureus*	*E. coli*	*B. subtilis*	*P. aeriginosa*
*Anaphalis virgata* Thoms.	Asteraceae	135	Fever, breathing problems, blisters	Herba	-	-	-	-
*Arnebia guttata *Bge.	Boraginaceae	145	Ear-ache	Radix	6 (E)	-	-	-
*Artemisia persica* Boiss.	Asteraceae	154	Non-descript stomach problems	Herba	1000 (E)	-	-	-
*Artemisia sieversiana* Willd.	Asteraceae	152	Treatment for maggot infected wounds (in animals)	Herba	-	-	-	-
*Betula chitralica* Browich	Betulaceae	36	Boils/blisters, decoction drunk for various diseases	Cortex	-	-	-	-
*Carum carvi* L.	Apiaceae	17	Fever, throat pain	Semen	500 (E)	-	-	-
*Delphinium brunonianum* Royle.	Ranunculaceae	113	Antibacterial, applied to wounds	Herba	-	-	-	-
*Descurainia sophia* (L.) Webb & Berth	Brassicaceae	151	Antibacterial decoction, powder blown into hurting throats, blisters	Semen	-	-	-	-
*Ephedra intermedia* Schrenk & Mey.	Ephedraceae	163	Mouthwash for toothache/periodontitis	Herba	500 (E)500 (W)	-	-	-
*Epilobium latifolium* L.	Onagraceae	136	Blisters	Herba	1000 (E)	-	-	-
*Hyoscyamus pusillus* L.	Solanaceae	30	Toothache	Semen	-	-	-	-
*Lagochilus cabulicus* Rech, f & Edelb s.l.	Lamiaceae	141	For animal with lung troubles	Herba	500 (E)			
*Mentha longifolia* (L.) Hudson	Lamiaceae	175	Fever, decoction drunk for various unwellness	Herba	-	-	-	-
*Nepeta parmiriensis* Franch.	Lamiaceae	125	Fever, nausea	Herba	-	-	-	-
					*S. aureus*	*E. coli*	*B. subtilis*	*P. aeriginosa*
*Onobrychis echidna* Lipsky	Fabaceae	32	Toothbrush	Radix	-	-	-	-
*Peganum harmala* L.	Zygophyllaceae	1	Childrens ear-ache, powder applied to blisters	Semen	500 (E)	500 (E)	500 (E)	-
*Plantago gentianoides subsp. Griffithii* (Dechne.) Reich.	Plantaginaceae	16	Blisters (with pus), absesses and wounds	Semen	-	-	-	-
*Rosa webbiana* Wallich.	Rosaceae	160	Fever, non-descript stomach problems. Decoction for bloody coughing, ashes for ear-ache	Fructus	-	-	-	-
*Rumex patientia subsp. pamiricus* Rech	Polygonaceae	161	Antibacterial, fever	Radix	1000 (E)	1000 (E)	750 (E)	1000 (E)
*Ziziphora clinopodioides* Lam	Lamiaceae	107	Fever	Herba	-	-	-	-
Streptomycin					2	6	6	6

(-): MIC values > 1,000 µg. (E): Ethanol extract, (W): water extract.

**Table 2:** COX-1 inhibitory effect of plant species used in the Pamir Mountains to treat ailments related to pain, fever and inflammation.

Plant species	Family	Voucher No.	Reported use	Used part	COX-1 inhibition (%)				
					Total assay concentration (µg/mL)				
					0.05	0.5	5	50	500
*Artemisia persica* Boiss.	Asteraceae	154	Non-descript stomach problems, headache, applied warm to swollen body parts, applied to chest for common cold	Folium + Flos	1	45	85	75	91
*Dragocephalum paulsenii *Briq.	Lamiaceae	51	Fever	Herba	−6	48	96	76	88
*Elsholtzia densa *Benth	Lamiaceae	147	Headache, joint pain, non-descript stomach problems	Herba	0	13	41	98	100
*Ephedra intermedia* Schrenk & Mey.	Ephedraceae	163	Bath for broken body-parts, poultice, swollen stomach, backache, bath for aching feet, mouthwash for toothache/periodontitis	Herba	−3	−9	78	96	103
*Hyoscyamus pusillus* L.	Solanaceae	30	Toothache	Semen	54	36	115	87	104
*Mentha longifolia* (L.) Hudson	Lamiaceae	175	Fever, backache, decoction drunk for various unwellness	Herba	30	−4	-	98	105
*Nepeta parmiriensis* Franch.	Lamiaceae	125	Fever	Herba	23	38	97	83	106
*Primula macrophylla *Don	Primulaceae	114	Eye pain	Dust from flowers	3	7	42	101	108
*Rumex patientia subsp. pamiricus* Rech	Polygonaceae	161	Anti-inflammatory, fever	Radix	5	11	52	73	86
*Ziziphora clinopodioides* Lam	Lamiaceae	107	Fever, headache	Herba	−25	−22	49	27	67

An *in vivo* study on *Ephedra intermedia* has shown that a methanol extract inhibited swelling in the carrageenan-induced paw edema assay [8]. Bioassay-guided isolation identified ephedroxane as the anti-inflammatory compound [9]. Water extracts of *Rumex patientia* have in previous studies shown anti-inflammatory activity in several paw-oedema models [10], and also exhibited analgesic effect in formaldehyde–induced pain [11]. Previous analysis of the essential oil of *Nepeta pamiriensis* collected in this study, showed that the oil contains 98% 1,8-cineole [12]. 1,8-Cineole has in several studies been shown to possess anti-inflammatory activity, including activity mediated via inhibition of the prostaglandin synthesis [13].

## 3. Experimental Section

### 3.1. Plant Material

Plants were collected during the summer season of 2010 in the Wakhan valley, Big and Small Pamir. Plant material was air dried out of sunlight and kept in paper bags. Voucher specimens were identified by Jens Soelberg and deposited at the Herbarium of The Botanical Museum of Copenhagen University (C) and Kabul University Faculty of Science Herbarium (KUFS). See Table 1 and Table 2 for voucher numbers.

### 3.2. Extraction for Antibacterial Assay

Dried, powdered material (1 g) of plant material was extracted with 3 mL of water or ethanol for 30 min in an ultrasound bath. The extract was filtered through a filter paper. The extraction procedure was repeated. After filtration the combined ethanol extract was evaporated to dryness under nitrogen, whereas the water extracts were freeze-dried. The extracts were redissolved in DMSO to 100 mg/mL and diluted with Mueller-Hinton broth to a final concentration of 8 mg/mL.

### 3.3. Extraction for COX-Assay

Dried, powdered material (100 mg) of plant material was extracted with 1 mL ethanol for 30 min in an ultrasound bath and filtered through a filter paper. The extraction procedure was repeated. After filtration the extract was evaporated to dryness under nitrogen and redissolved in ethanol to a final concentration of 40 mg/mL.

### 3.4. Antibacterial Assay

The antibacterial assay was performed in 96-well microplates. Bacteria (*Staphylococcus aureus* ATCC 6538; *Eschericia coli* ATCC 11229; *Bacillus subtilis* ATCC 6633; *Pseudomonas aeriginosa* ATCC 9027) were cultured overnight in Mueller-Hinton broth at 37 °C. 100 µL overnight culture was added to 9.9 mL Mueller-Hinton broth. Each well contained 50 µL test solution (plant extract, streptomycin or broth), 50 µL broth and 100 µL bacterial suspension. The plates were incubated overnight at 37 °C. After incubation, 40 µL MTT (3-(4,5-dimethylthiazol-2-yl)-2,5-diphenyltetrazolium bromide) was added to each well and the plate was incubated for 30 min before observation for blue color formation.

### 3.5. Cyclooxygenase-1 Assay

The COX-1 assay was performed according to [14] with minor modifications. Fifty µL of COX-1 (Sigma) (75 unit per sample) and 1,250 µL co-factor solution (0.003 g *l*-adrenaline, 0.003 g reduced gluthatione) and 200 µL Tris-buffer per sample were preincubated for 15 min on ice. Sixty µL of this solution was added to the test solution consisting of 2.5 µL plant extract and 17.5 µL water and preincubated for 10 min at room temperature. ^14^C-Arachidonic acid (20 µL) was added to this enzyme-extract mixture and incubated for exactly 10 min in a water bath at 37 °C. The reaction was terminated with 10 µL 2 N HCl. In each test, two types of controls were run (2.5 µL ethanol and 17.5 µL water): backgrounds in which the enzyme was inactivated with HCl before the addition of ^14^C-arachidonic acid; and solvent blanks. The COX-1 inhibitor indomethacin was used as a positive control.

Unlabeled prostaglandin carrier solution (4 µL per sample) was added to the reaction mixture. ^14^C-prostaglandins synthesized in the assay were separated from unmetabolized arachidonic acid by column chromatography using silica columns. The assay mixture was applied to the column with 1 mL eluent 1 (hexane:1,4-dioxane:acetic acid (350:150:1 v/v/v)) followed by an additional 4 mL eluent 1 to elute the unreacted arachidonic acid. The prostaglandins were eluted into scintillation vials using 3 mL eluent 2 (ethyl acetate:methanol (85:15 v/v)). Four milliliters scintillation fluid (Pico-Flour 15, Perkin Elmer) was added to the vials and the radioactivity was counted after 1 h in the dark in a TriCarb scintillation counter. The percentage inhibition of the extracts was obtained by measuring the amount of radioactivity in the solutions relative to the solvent blank.

The assay was performed in triplicate. Data were fitted into Grafit5 software for estimation of IC_50_-values.

## 4. Conclusions

*Ephedra intermedia*, *Lagochilus cabulicus*, *Peganum harmala* and especially *Arnebia guttata* inhibited *Staphylococcus aureus*, and the *P. harmala* extract further inhibited the growth of *E. coli* and *Bacillus subtilis*. *Artemisia persica*, *Dragocephalum paulsenii*, *Ephedra intermedia*, *Hyoscyamus pusillus*, *Nepeta parmiriensis* and *Rumex patientia* subsp. *pamiricus* exhibited COX-1 inhibitory activity.

The results indicate that some of the plant species used in traditional medicine in the Pamir Mountains have *in vitro* activities that support their use.
